# The Contribution of Genetic Testing in Optimizing Therapy for Patients with Recurrent Depressive Disorder

**DOI:** 10.3390/clinpract14030056

**Published:** 2024-04-23

**Authors:** Rita Ioana Platona, Florica Voiță-Mekeres, Cristina Tudoran, Mariana Tudoran, Virgil Radu Enătescu

**Affiliations:** 1 Doctoral School, University of Medicine and Pharmacy “Victor Babes” Timisoara, 300041 Timisoara, Romania; platona.rita@umft.ro; 2 Psychiatry Department, County Clinical Emergency Hospital of Oradea, 410169 Oradea, Romania; 3 Morphological Disciplines Department, Faculty of Medicine and Pharmacy, University of Oradea, 410087 Oradea, Romania; 4 Department VII, Internal Medicine II, Discipline of Cardiology, University of Medicine and Pharmacy “Victor Babes” Timisoara, E. Murgu Square, No. 2, 300041 Timisoara, Romania; tudoran.cristina@umft.ro (C.T.); mariana.tudoran@gmail.com (M.T.); 5 Center of Molecular Research in Nephrology and Vascular Disease, University of Medicine and Pharmacy “Victor Babes” Timisoara, E. Murgu Square, No. 2, 300041 Timisoara, Romania; 6 Cardiology Clinic, County Emergency Hospital “Pius Brinzeu”, L. Rebreanu, No. 156, 300723 Timisoara, Romania; enatescu.virgil@umft.ro; 7 Discipline of Psychiatry, Department of Neurosciences, University of Medicine and Pharmacy “Victor Babes” Timisoara, 300041 Timişoara, Romania

**Keywords:** major depressive disorder, recurrent depressive disorder, scales for assessing the intensity and evolution of depression, genetic testing, pharmacogenetic-guided therapy

## Abstract

(1) Background: The aim of this study was to analyze the impact of pharmacogenetic-guided antidepressant therapy on the 12-month evolution of the intensity of depressive symptoms in patients with recurrent depressive disorder (RDD) in comparison to a control group of depressive subjects who were treated conventionally. (2) Methods: This prospective longitudinal study was conducted between 2019 and 2022, and the patients were evaluated by employing the Hamilton Depression Rating Scale (HAM-D), Hamilton Anxiety Rating Scale (HAM-A) and the Clinical Global Impressions Scale: Severity and Improvement. We followed them up at 1, 3, 6, and 12 months. (3) Results: Of the 76 patients with RDD, 37 were tested genetically (Group A) and 39 were not (Group B). Although the patients from Group A had statistically significantly more severe MDD at baseline than those from Group B (*p* < 0.001), by adjusting their therapy according to the genetic testing, they had a progressive and more substantial reduction in the severity of RDD symptoms [F = 74.334; η^2^ = 0.674; *p* < 0.001], indicating a substantial association with the results provided by the genetic testing (67.4%). (4) Conclusions: In patients with RDD and a poor response to antidepressant therapy, pharmacogenetic testing allows for treatment adjustment, resulting in a constant and superior reduction in the intensity of depression and anxiety symptoms.

## 1. Introduction

Major depressive disorder (MDD), with its large spectrum of clinical manifestations, represents an important public health concern due to its increased prevalence and high recurrence tendency, resulting in disability and a reduced quality of life (QoL), in addition to its subsequent elevated socioeconomic costs [1,2].

Currently, MDD constitutes one of the principal domains of clinical psychiatry and mental health research, attracting high levels of interest from mental health professionals and the wider medical community. This attitude is explained by the epidemiological reality, considering the increased prevalence and incidence of MDD in the adult population. This is also confirmed by official World Health Organization (WHO) reports stating that, in the year 2020, MDD represented the second-leading cause of disability among all potentially invalidating diseases worldwide. Additionally, MDD implies increased costs (direct, but especially indirect) determined by its treatments, considering that MDD represents one of the most treatable mental disorders [3]. Direct medical costs are related to diagnosis, evaluation, hospitalization, and both medical and nonmedical treatment, including prevention and rehabilitation of patients with MDD, while the indirect costs result from the incapacity of people suffering from MDD to properly perform their profession, frequently resulting in impaired quality and productivity of their work or even unemployment [4]. Other indirect costs are consequences of their temporary or long-term disability, sometimes resulting in premature mortality due to healthcare neglect and increased suicide rates encountered in patients with MDD, frequently with economic consequences on their families who have to take care of these patients [4,5,6]. Moreover, MDD is frequently associated with other medical conditions that could potentially aggravate depressive symptoms and prolong the duration of hospitalization and disability related to depression in these patients [7,8]. On the other hand, MDD negatively impacts the evolution and therapy of other diseases, especially cardiovascular diseases, even facilitating the development of some cardiovascular dysfunctions [9]. Unfortunately, the prophylaxis and precocious diagnosis of MDD in the general population, especially at the level of primary medical assistance, is still strongly influenced by the patient’s socioeconomic level [10].

Frequently, MDD is accompanied by symptoms of anxiety, especially in the elderly, among whom depression must be treated as a priority. If MDD occurs in a patient already diagnosed with an anxious disorder, this pathology needs to be treated first [11,12].

It is well known that MDD manifests a high tendency toward recurrence, so the term recurrent depressive disorder (RDD) defines MDD by recurrent episodes, characterized by the occurrence of two or more episodes of depressive symptoms separated by periods of remission within a year [1]. Another term, resistant depression (RD) or treatment-resistant depression (TRD), established by the European Medicines Agency (EMA), is considered if an adequate therapeutic response has not been achieved after therapy with at least two antidepressants from different pharmacological classes, in appropriate therapeutic doses, and for a sufficient period of time (minimum of 6 weeks) [13]. It is considered that approximately 40% of all patients with MDD manifest RDD, with persistent depressive symptoms, sleep disorders, fatigue, and recurrent thoughts about death. It has been observed that the elderly, women, and people with other pathologies are more vulnerable to developing RDD [14].

To quantify the intensity of depressive symptoms, the severity of accompanying anxious elements, and their evolution under therapy, several instruments have been developed, such as the Hamilton Depression Rating Scale (HAM-D) and Hamilton Anxiety Rating Scale (HAM-A), created by Hamilton in 1960, which was one of the first scales but is still among the most used, both in clinical practice and in research [15,16]. Its initial purpose was to assess the severity of MDD episodes. Although it was not designed for diagnostic purposes, it is frequently used in this sense in clinical research that employs threshold scores to indicate the presence of a depressive episode. Other scales, such as the Clinical Global Impressions Scale with its two domains (severity and improvement (CGI-S, CGI-I)), are clinical instruments that have been developed by the National Institute of Mental Health (1976) to follow up on the evolution of MDD [17].

Several antidepressant drugs have been developed, starting from the hypothesis of the hypofunction of aminergic brain transmission, e.g., serotonergic, noradrenergic, and dopaminergic circuits. From this perspective, some drugs act selectively on one neurotransmission system, while others act more or less selectively on several neurotransmission systems (new-generation antidepressants versus classical antidepressants) [7,8]. Depending on the clinical effectiveness, costs, and implementation factors, the Nice Guidelines 2022 [18] recommend combining antidepressant treatment with cognitive behavioral therapy (CBT), group exercise or guided self-help, interpersonal psychotherapy (IPT), short-term psychodynamic psychotherapy (STPP), or individual behavioral activation (BA). The choice of treatment takes into account the specific effects of the drugs, the risk of suicide, and the history of response to antidepressant drugs. For first-line therapy, selective serotonin reuptake inhibitors (SSRIs), serotonin–norepinephrine reuptake inhibitors (SNRIs), or tricyclic antidepressants (TCAs) are recommended. Other antidepressants could be indicated based on the patient’s clinical history and previous treatment. SSRIs are generally well tolerated, have a good safety profile, and should be considered as the first choice for most people [19,20,21]. Antidepressant treatment should be taken for at least 6 months, and the benefits should be felt in 4 weeks [18].

Since RDD and TRD are quite frequent and therapeutic failure or partial recovery is not unusual, new methods to assess the mechanisms and causes of impaired therapeutic response have been elaborated. Genetic testing offers major advantages to healthcare professionals, providing them with genetic information that may help them to select appropriate medications for individuals with mental illnesses and other brain disorders. In this direction, the Genomind Professional PGx genetic test has been developed to provide data that may help healthcare professionals to optimize drug therapy for patients with mental health disorders. This genetic test provides important information about the patient’s genetic profile by analyzing 24 genes involved in the patient’s response to 130 drugs employed in the treatment of depression, anxiety, bipolar disorder, schizophrenia, autism, attention deficit hyperactivity disorder, posttraumatic stress, obsessive–compulsive disorder, addiction and substance abuse, and chronic pain. Developed in May 2022, the new updated version of the Genomind genetic test offers a personalized approach to the management of mental disorders, recognizing the variation in treatment effectiveness and the sensitivity of mental health disorders induced by genetic changes. Thus, the pharmacogenetic test improves the therapeutic results for patients through a more precise selection of the drugs that are suitable for the patient according to their genetic material [22,23].

A limited number of studies are available in the specialized literature concerning the clinical impact of genetic testing on the selection of an adequate antidepressant therapy according to the patient’s pharmacogenetic profile [24,25]. Therefore, according to the more recent data debated in the medical literature [26], we strongly consider that further clinical studies are required concerning the possibilities offered by genetic testing for appropriate pharmacogenetic-guided therapy in patients with depression and anxiety.

The aim of this study was to analyze the differences concerning the 12-month evolution of the intensity of RDD symptoms in patients for whom treatment was optimized in accordance with information offered by genetic testing in comparison to a control group of subjects who did not benefit from this testing.

## 2. Materials and Methods

### 2.1. Study Population

Between January 2019 and December 2022, we conducted a prospective longitudinal study in the PsihoNeuroMag Clinic in Oradea, a city in the northwest of Romania, on a study population consisting of 76 patients already diagnosed with RDD, with at least 2 recurrences in the last two years, and with persistent symptoms despite the current therapy. They were selected based on the inclusion/exclusion criteria from all patients with RDD who attended the clinic in this period for a new episode of depression despite therapy and who agreed to participate in the study. All the patients were informed that their participation in the study was voluntary, that they could leave the study at any time without consequences, and that the researchers guaranteed the privacy and confidentiality of the collected data. All those aged under 18 years, those unable to understand and sign an informed consent form, those with cognitive impairments, subjects who did not understand Romanian, and those who refused to participate were excluded from the study. The subjects also underwent an assessment of their cognitive level using the Mini-Mental State Examination (MMSE) scale [27], and subjects with a score under 24 (suggesting dementia) were excluded as candidates for this study, since cognitive impairment is frequently associated with depression. After agreeing to participate in this study, the patients’ medical history, baseline data, and current medication were collected, and the intensity of depressive symptoms and of the associated anxiety were assessed by employing the HAM-D, HAM-A, and CGI-S questionnaires. The intensity of their mental health complaints was assessed by the same skilled senior specialist psychiatrist who also informed them regarding the therapeutic possibilities and the advantages offered by the pharmacogenetic-guided therapy. All the patients included in the study were recommended to undergo genetic testing via the Genomind Professional PGx test to obtain additional data that could allow for the adjustment of their antidepressant therapy, but only 37 of them agreed, while the other 39 refused for various reasons, although they were offered the same explanations by the same psychiatrist. According to this criterion, our study population was divided into two groups: Group A, including 37 patients who agreed to undergo genetic testing for establishing pharmacokinetic and pharmacodynamic variations; and Group B, consisting of 39 individuals who did not agree to such testing. Depending on the results provided by the genetic testing, the treatment was individualized for each patient from Group A, while for those from Group B, the therapy was adjusted empirically according to the guideline recommendations [18]. Therefore, in Group A, there were four main therapeutic approaches: (1) initial antidepressant class maintained with an increased dose; (2) replacement of the previous treatment with another class; (3) initial antidepressant class maintained with the addition of a neuroleptic; and (4) another antidepressant associated with a neuroleptic/thymostabilizer.

The evolution of patients from both groups was followed over time (longitudinally), and all modifications of the intensity of their symptoms observed using HAM-D and HAM-A were noted at their inclusion (T1) and after 1 month (T2), 3 months (T3), 6 months (T4), and 12 months (T5). Furthermore, the general assessment of the current severity of the patient’s symptoms was measured in the inclusion stage using the CGI-S: a tool that is used in psychiatry and applied by the observer. The CGI has proven over time to be a robust measure of efficacy in many clinical trials and is easy to administer [27,28,29,30,31]. In the later stages, i.e., T2–T5, the CGI-I improvement scale was used to track the patients’ progress.

In the third part of the longitudinal study, the results of the patients for the CGI-S were recorded in the inclusion phase (T1), and the results for the CGI-I were recorded after 1 month (T2), 3 months (T3), 6 months (T4), and 12 months (T5).

Ethical approval for this research was obtained from the PsihoNeuro Mag Clinic, Oradea, Romania (22/12.03.2019, approved on 12 March 2019).

### 2.2. Assessment of Depressive/Anxious Symptoms

(a) HAM-D was elaborated by Hamilton (1960), and although the initial version included 21 items, the current version contains 17 items investigating various domains such as depersonalization/derealization, paranoid and obsessive symptoms, diurnal mood variations, etc. The scales are applied by the physician, the required time is approximately 20 min, and all the obtained information is employed to evaluate the patient. The majority of the items are quoted on a Likert scale from 0 to 4, but several items (referring to insomnia, somatic symptoms such as gastrointestinal, genital, and general symptoms, and weight loss) are quoted from 0 to 2. The total score may vary from 0 to 50, where a total score equal to or lower than 7 is considered normal, a score ranging from 8 to 13 signifies mild depression, a score ranging from 14 to 18 indicates moderate depression, a score ranging from 19 to 22 shows severe depression, and scores equal to or higher than 23 indicate very severe depression [15].

(b) HAM-A is the section of Hamilton’s scale employed to evaluate anxiety, and it quantifies the intensity and severity of anxious symptoms. It is a 14-element questionnaire designed to evaluate various perceptions such as pressure, fear, insomnia, mood, depression, various somatic symptoms (sensory, cardiovascular, respiratory, gastrointestinal, genitourinary, etc.), and behavioral attitudes observed during the interview. Each element is quoted from 0 (not present) to 4 (severe); a total over 17 (out of a possible 56) indicates mild anxiety, while a score from 25 to 30 is considered to indicate a moderate–severe episode [16].

(c) The Clinical Global Impressions (CGI) scale refers initially to three different global measurements: the severity of the disease (CGI-S), the global improvement (CGI-I), and the efficiency index (CGI-E). The CGI-S score generally follows the CGI-I score; therefore, the improvement of one of them is accompanied by an improvement in the other [17,28,29,30,31,32].

### 2.3. Genetic Testing

The patients who agreed to undergo the genetic testing were scheduled for this exploration after they were informed about the procedure and the benefits of this testing. The genetic test Genomind Professional PGx, which is the most complex pharmacogenetic test available, was employed. A special kit was employed to scrape a sample from the oral cavity, and the collected probe was sent to the medical laboratories of Genomind, INC. (King of Prussia, PA, USA). Each probe was tested twice to maintain a 99.9% precision of the obtained results. These results offered important information on the patient’s genetic profile by analyzing polymorphisms in 24 genes implicated in the subject’s response to 130 drugs employed in the treatment of affective disorders, psychosis, chronic pain, and drug abuse. Of the 24 genes analyzed using the Genomind Professional PGx test, 15 refer to drugs’ pharmacodynamics (indicating their interactions with receptors, transporters, and neurotransmitters), thus allowing for the determination of how appropriate the drug is for the specific patient, while the other 9 are pharmacokinetic genes (useful for establishing the appropriate dose) [23]. This testing is an important method to establish a personalized therapy for each patient depending on their genetic profile, with adequate medication classes and adjusted doses, especially for those with a partial therapeutic response, with short periods of remission and frequent relapses. According to this testing, the treatment of patients from Group A was adjusted by replacing the previous antidepressant drug, by increasing the dose, or by adding another class of medication, while in Group B, where we did not benefit from the results of the genetic testing, therapy was adjusted empirically.

### 2.4. Statistical Methods

The statistical data were processed using SPSS version 22. Numeric variables were presented as the mean and standard deviation (SD) or as the median and interquartile range (IQR), while categorical variables were presented as frequencies and percentages. We employed the Shapiro–Wilk test to check the numeric variables for Gaussian distribution. In the first part of the research, we employed the *t*-test and χ2 test to analyze demographic data in Groups A and B and the results of different depression scales. 

The design of this research was longitudinal, and we were mostly interested in the impact and effectiveness of adjusting the medication according to the genetic testing with respect to the evolution of the intensity of depressive symptoms, as quantified by the HAM-D, as well as that of anxiety, as measured by the HAM-A.

In the second part of the analysis, we employed the ANOVA unifactorial test at specific intervals, with the intensity of depression and anxiety used as dependent variables. The results were considered statistically significant if *p* < 0.05.

In the third part of the statistical analysis, we studied the influence of adapting the therapy for the genetically tested patients based on the evolution of depression severity, as measured by the CGI-S. From a conceptual point of view, the notion of improvement refers to the clinical distance between the current state of the patient and that before the beginning of the therapy or during the course of the treatment. To analyze the impact of adjusting the treatment according to the results of the genetic testing on the intensity of depressive symptoms, we used a one-factor ANOVA with repeated measurements. The experimental design was 1 × 2, where the independent variables were the type of disease (RDD) and the adjustment of medication according to the genetic testing (genetically tested patients versus untested patients), while the dependent variable was represented by the CGI-S scores, considering that both patient groups were tested at five distinct stages of symptom severity (T1–T5), as measured by CGI-S at T1 and by CGI-I between T2 and T5. We repeated the ANOVA measures using the results of the CGI-S and CGI-I as the dependent variables. The results were considered significant at *p* < 0.05.

## 3. Results

### 3.1. Baseline Characteristics of the Study Groups

The total group of patients with RDD included 76 individuals, aged between 18 and 70 years (mean age: 43.13 ± 15.10 years), of whom 26 (34.2%) were men and 50 (65.8%) were women. Concerning the patients’ educational levels, the patients with RDD had higher levels of education: only 1 (1.3%) had failed to complete secondary school, 46 participants (60.5%) were high school graduates, and 29 participants (38.2%) had received higher education. Regarding the marital status of our subjects with RDD, 35 were unmarried (46.1%), 33 were married (43.4%), 3 were divorced (3.9%), 4 were living in a cohabiting relationship (5.3%), and 1 was widowed (1.3%). Most of the participants (41; 53.9%) were employees, only 26 subjects (34.2%) were retired, and there were 2 (2.6%) housewives and 7 (9.2%) students. Seventy participants (92.1%) declared an average income, five (6.6%) declared a high income, and only one subject (1.3%) declared a low income (Table 1).

Regarding their place of origin, 26 of the participants (34.2%) were from rural areas, while 50 of the participants (65.8%) were from urban areas. From an ethnic point of view, there were 70 Romanians (92.1%) and 6 Hungarians (7.9%) (Table 1). It should be mentioned that except for the levels of education (high school or college) and urban residence, which were significantly higher in Group A (*p* = 0.003 and *p* = 0.02, respectively), there were no statistically significant differences between groups A and B.

### 3.2. Results Concerning the Assessment of MDD Severity and Evolution

**Figure 1:**
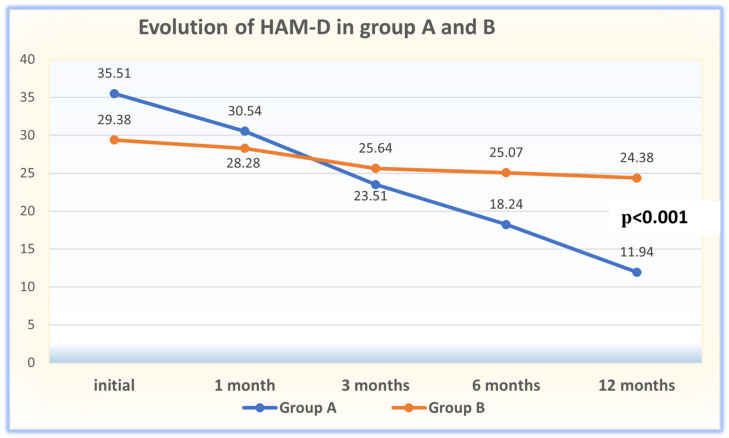
Evolution of RD intensity assessed in the patient groups. Legend: HAM-D = Hamilton Rating Scale for Depression.

**Figure 2:**
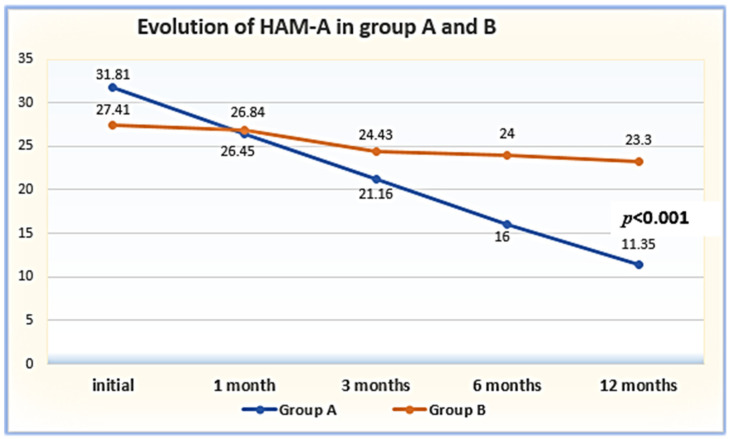
Evolution of RD intensity assessed in the patient groups. Legend: HAM-A = Hamilton Rating Scale for Anxiety.

**Figure 3:**
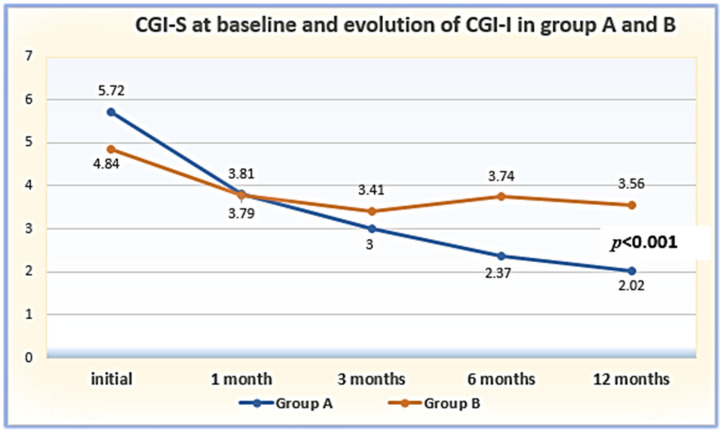
Graphical representation of CGI-I evolution in the patient groups.

The results of the initial evaluation using the HAM-D, HAM-A, and CGI-S scales, their evolution at 1, 3, 6, and 12 months, and the results of the CGI-I assessment are presented in Table 2. As can be observed, the patients from Group A had statistically significantly worse results at baseline across all the scales (*p* = 0.001). The evaluation offered by all the scales indicated a significant improvement in both groups, but this was much more evident in Group A, such that the HAM-D scores overlapped between months 1 and 3, while those assessed using HAM-A and CGI-I already overlapped at month 1 (Table 2; Figure 1, Figure 2 and Figure 3).

According to the unifactorial ANOVA, in the patients with RDD from Group A who were evaluated between T1 and T5 with HAM-D [F = 74.334; *p* < 0.001; η^2^ = 0.674], a significant (Table 3) and constant (Table 4; Figure 1) reduction in the severity of depression was observed, indicating a substantial association with the genetic testing (67.4%). Meanwhile, in the patients with RDD who did not benefit from the results of the genetic testing, the results of the HAM-D scale [F = 21,218; *p* < 0.001; η^2^ = 0.358] indicated a fluctuating reduction (Figure 1), with the efficiency of the therapy being moderate.

### 3.3. Results Concerning the Evolution of Anxiety Levels in Patients with MDD

Another aspect of evaluating the patients with RDD using the Hamilton Scale was testing their anxiety levels using the HAM-A component at baseline (T1) and beyond. The results, expressed as mean values and standard deviations, are presented in Table 2 and Figure 2. The statistical analysis of the patients’ average HAM-A scores indicated more severe anxiety at baseline in Group A (m = 30.78; SD = 5.90), which decreased continuously under therapy, resulting in a significant reduction at 12 months (m = 11.10; 5.45), corresponding to moderate anxiety. In contrast, the patients from Group B, although they were less anxious at baseline (m = 26.92; AS = 4.20), had a less favorable 12-month evolution (m = 24.46; SD = 4.07) (see Table 5).

By employing ANOVA testing, we observed that the patients from Group A had a more favorable progression between T1 and T5 in terms of their HAM-A scores [F = 160.621; *p* < 0.001; η^2^ = 0.817], with a significant (Table 5) and constant reduction (Table 5; Figure 2) in their anxiety levels, indicating a substantial impact of the adjusted therapeutic approach (81.7%). Considering the patients with RDD from Group B, the HAM-A results [F = 18.875; *p* < 0.001; η^2^ = 0.332] indicated an oscillating reduction (Figure 2).

The differences registered between the various stages of testing are depicted in Table 6.

### 3.4. Longitudinal Changes in CGI Parameters in Group A and B Patients

The analysis of means on the CGI-S scale in the inclusion phase indicated statistically significant differences between the genetically tested depressed patients and the non-genetically tested patients (t = 5.649; *p* < 0.001).

CGI-S was utilized at baseline (see Table 2 and Figure 3). Regarding the patients’ follow-up, CGI proved to offer a robust quantification of efficiency over time, and because it is easy to administer, it has been utilized in many clinical studies [27,28,29,30,31]. Subsequently, at visits T2–T5, we employed the improvement scale (CGI-I) to follow up on the patients’ progress. In the third part of the study, we registered the patients’ results offered by the CGI-S scale at inclusion, and those provided by the CGI-I testing were registered at 1 month (T2), 3 months (T3), 6 months (T4), and 12 months (T5) (Figure 3). The statistical analysis of the means provided at baseline using the CGI-S scale indicated statistically significant differences between the patients from Group A and those from Group B (t = 5.649; *p* < 0.001).

## 4. Discussion

MDD is a complex disorder that has been considered for more than two decades to have a background of increased heritability, reaching as high as 37% according to the estimates of two twin studies [33,34,35,36]. Despite strong evidence for a genetic component, identifying the specific gene variants responsible for the development of this disorder has always constituted a major challenge. Genome-wide association studies have tested the existence of differences in the allele frequencies between patients with MDD and control groups, with millions of common single-nucleotide polymorphisms throughout the genome [34]. These differences may be functionally relevant for this disease, or they may indicate loci that are transmitted in a linkage disequilibrium with a causative polymorphism [35].

In the domain of psychiatric nosology and therapy, depressive disorder and anxiety have a long and close common history. Analyses of large-scale epidemiological surveys have identified major patterns of phenomenological overlap between these two conditions. Over time, the hypothesis of a common genetic background has been tested as a potential basis for this relationship. A previous family study by Hettema et al. [36] debated evidence concerning the co-occurrence of anxiety and MDD, while twin studies indicate shared genetic risk factors that could potentially explain these comorbidities [32,33,36]. Therefore, studying the molecular genetics of these pathologies can potentially provide support for specific genetic loci that could influence individuals’ susceptibility to developing a spectrum of depressive and/or anxious symptoms [37,38].

In our study, all the patients had both RDD and anxiety at baseline, as diagnosed using the HAM-D and HAM-A scales, but the intensity of the symptoms was more severe in the group of patients who accepted genetic testing (Group A). These patients were exasperated by the severity and persistence of their depressive symptoms, had generally higher levels of education, and understood the importance of the genetic testing for optimizing their treatment, since previous therapies had failed. Afterwards, we focused our statistical analyses on the longitudinal changes in these symptoms between T1 and T5, mainly concerning the intensity of depressive symptoms, but also considering the levels of anxiety as a consequence of treatment adjustments implemented based on the genetic testing. As discussed above, the patients who benefited from the results of this testing had a gradual and constant improvement in the severity of their symptoms, in comparison to those who were treated empirically and experienced a more modest evolution. We started from the premise that the progressive temporal reduction in the patients’ HAM-D results in Group A was a consequence of adjusting their treatments based on the genetic testing, as had clearly already occurred after the first month of guided therapy, improving further at the subsequent visits. From a psychometric point of view, it was highlighted that in patients from Group A, who had more severe RDD at baseline (as assessed by the HAM-D scores) compared to Group B (*p* ˂ 0.001), a significant decrease in RDD severity was achieved, from “high” at inclusion to “mild” after 12 months, while in Group B, the severity of depression had decreased less substantially at 12 months. Another aim of our study was to examine the evolution of the anxiety levels in the genetically tested versus non-genetically tested patients by employing a similar design as in the case of depressive symptoms. The analysis of the HAM-A results, from a psychometric point of view, indicated that, in Group A, a decrease in the mean levels of anxious symptoms was achieved, from “severe” at inclusion to “moderate” after 12 months (associated with the genetic testing in a proportion of 81.7%), while in Group B, the baseline anxiety levels had decreased to a lesser extent after 12 months, indicating reduced efficacy of the treatment.

There are discrepancies among the results of various studies on the psychometric performance of the CGI assessment; therefore, further research is needed. Specifically, there are controversies regarding the extent to which CGI testing provides a valid measure of the patient’s status and, if so, whether it is more appropriate to use the CGI-I or a differential score offered by the CGI-S as an outcome criterion [28,29]. This is why, in our longitudinal study, we evaluated the temporal improvements in the patients’ conditions using the CGI-S scale upon their inclusion in the study, while for the evaluation after one year, we employed the CGI-I scale. There were statistically significant differences in the CGI-S scores between Groups A and B at the beginning of the study, classifying the patients with RDD from Group A as being in the “marked sick” category, while those from Group B fell into the “moderately ill” category. The CGI-I scale was applied during the follow-up stages of the research (between T2 (one month) and T5 (one year)) after adjusting the treatment, with the patients’ health and general clinical status being recorded. By following the modifications in the CGI-I scores over the course of one year in Groups A and B, the data analysis indicated the greatest improvement in the condition of the patients from Group A. In Group B, the CGI-I scores indicated an improvement in the first 3 months, followed by a return to baseline. The analysis of interactions between RDD and pharmacogenetic-guided therapy indicated statistically and clinically significant results.

In a pharmacogenomic meta-analysis published by Brown et al. [39] in 2022 and in a randomized, controlled, participant- and rater-blinded trial published by Perlis et al. (2020), it was concluded that pharmacogenomic-guided antidepressant therapy is associated with a modest but significant increase in the remission of depressive symptoms in adults with MDD [26]. In our study, we obtained similar results: our data indicated a constant reduction in the severity of depressive and anxious symptoms, indicating a substantial association with the therapy adjustment guided by the genetic testing (67.4%).

Another observational study [40] that evaluated the effectiveness of genetic testing in optimizing treatment for patients with mental health disorders highlighted that over 80% of patients who benefited from genetic testing reported significant improvements, reduced adverse effects of drugs, improved quality of life, and significantly lower treatment failure rates. According to this research, the employment of pharmacogenetic tests to guide therapeutic choices is recommendable and even mandatory to guide treatment for MDD and/or anxiety, especially when the patient’s previous treatment has failed [39]. The works of Wood et al. [41] and King et al. [42] support these arguments, showing similar results (but in a different research line) concerning the beneficial effects of drug optimization based on PGx testing, resulting in the alleviation of symptoms in depressive patients (F(1.63, 45) = 5.45, *p* = 0.01, η^2^ = 0.11) along with mental health improvements (F(2.00, 45) = 4.16, *p* < 0.05, η^2^ = 0.16) and fewer side effects.

Furthermore, the Genomind testing results and treatment recommendations, along with the clinical guideline recommendations provided by organizations such as the Drug–Gene Pair Working Group (DWPG) and the Clinical Pharmacogenetics Implementation Consortium (CPIC), recommend PGx testing, since genetic variation may influence the recommended dose, efficiency, and safety profile of various antidepressants. For example, CYP2D6, CYP2C19, and CYP2B6 strongly influence the metabolism of several antidepressant drugs [40,41,42]. Moreover, pharmacodynamic genes can be used to explore the efficacy and side effects of these drugs. The aim of the Clinical Pharmacogenetics Implementation Consortium (CPIC) is to update and extend the indications of pharmacogenetic testing [41]. In 2021, Brouwer et al. published the Dutch Pharmacogenetics Working Group (DPWG) guidelines, which present the gene–drug interactions for the genes CYP2C19 and CYP2D6 and SSRIs, concluding that genotyping should be recommended to each patient before starting antidepressant therapy [24,25,43].

Decreasing the levels of depression and the burden of this costly disease is a priority; however, limited understanding of the biological basis of depression has hindered the development of new treatments and interventions. As there are currently a limited number of clinical studies on the importance of pharmacogenetic testing for the management of depression and anxiety, further investigation is required.

Our study refers to a current topic, the treatment of patients with TRD, a subset of RDD, for which pharmacogenetic-guided antidepressant therapy offers the perspective of a personalized prescription, which is more efficient and has fewer adverse effects. This study is a prospective clinical study, one of the first in our country that analyzes the clinical impact of PGx on the management of patients with TRD. One of the main qualities of our research is its longitudinal design, allowing the follow-up of patients’ evolution for a year by using several psychometric scales to evaluate the intensity of depression and anxiety symptoms at five specific intervals starting from baseline and after optimizing the treatment. The information offered by our research could be employed to elaborate an algorithm for personalizing the management of patients with TRD according to the results of the genetic testing.

This study has some limitations, particularly resulting from the fact that it was a single-center study conducted on a limited number of patients. Another aspect results from the repeated application of the same scales, which could have led to the subjects learning the answers and to an increased degree of subjectivity when answering the items. Although during the selection process we did not take into consideration possible education discrepancies between the study participants, and the same information about the utility of genetic testing was offered by the same psychiatrist to all of them, an unforeseen finding of our study was that the subjects with higher education levels and from urban areas were more prone to accept this investigation. It should be mentioned that they also had higher levels of depression and anxiety at the beginning of the study, but they also had greater benefits from the treatment optimization. Finally, we cannot forget that this study was conducted during the COVID-19 pandemic, when individuals, especially from urban agglomerations, were more prone to depressive disorders and had higher anxiety levels [44,45,46].

## 5. Conclusions

In this study, we found that pharmacogenetic testing allowed for the efficient adjustment of treatment in patients with severe and recurrent depressive disorder and a poor response to antidepressant therapy, resulting in a progressive reduction in the intensity of depressive and anxious symptoms. This genetic testing thus allows for the better management of treatment, even in patients with more severe depressive symptoms, in comparison to therapeutic attitudes guided by the physician’s experience alone. Therefore, genetic testing should be recommended to a greater extent in order to ameliorate the evolution of symptoms in patients suffering from depressive illnesses while also enabling a faster recovery.

## Figures and Tables

**Table 1:** Sociodemographic characteristics of patients from groups A and B.

Characteristics		Total Sample = 76 Subjects (No./Percent)	Group A = 37 Subjects Genetically Tested (No./Percent)	Group B = 39 Subjects Not Tested (No./Percent)	Chi^2^	*p*
Gender:	Men	26 (34.2%)	16/43.2%	10/25.6%	0.791	0.373
	Women	50 (65.8%)	21/56.8%	29/74.4%	1.672	0.195
Education:	˂4 grades	0	0	0	0	0
	≥8 grades	1 (1.3%)	0	1/2.6%	0	0
	High school	46 (60.5%)	14/37.8%	32/82.1%	8.683	0.003 *
	College	29 (38.2%)	23/62.2%	6/15.4%	4.035	0.04 *
Civil status:	Single	35 (46.1%)	21/56.8%	14/35.9%	1.427	0.232
	Married	33 (43.4%)	14/37.8%	19/48.7%	0.377	0.539
	Divorced	3 (3.9%)	1/2.7%	2/5.1%	0.006	0.937
	Stable relationship	4 (5.3%)	1/2.7%	3/7.7%	0.023	0.878
	Widowed	1 (1.3%)	0	1/2.6%	0	0
Occupation:	Employed	41 (53.9%)	21/56.8%	20/51.3%	0.122	0.727
	Retired	26 (34.2%)	9/24.3%	17/43.6%	0.905	0.341
	Unemployed	2 (2.6%)	0	2/5.1%	0	0
	Student	7 (9.2%)	7/18.9%	0	0	0
Provenience:	Urban	26 (34.2%)	30/81.1%	20/51.3%	4.898	0.02 *
	Rural	50 (65.8%)	7/18.9%	19/48.7%	1.810	0.178

Legend: * statistically significant, *p* < 0.05.

**Table 2:** Results of HAM-D and HAM-A testing at baseline and their evolution.

Scales Employed		Group A = 37 Subjects with Genetic Testing (m/SD)	Group B = 39 Subjects Not Tested (m/SD)	T	*p*
HAM-D:	initial (T1)	35.51 ± 6.87	29.38 ± 3.87	4.821	0.001 *
	1 month (T2)	30.54 ± 11.88	28.28 ± 3.47	1.137	0.259
	3 months (T3)	23.51 ± 11.93	25.64 ± 3.68	−1.062	0.292
	6 months (T4)	18.24 ± 11.94	25.07 ± 3.01	−3.460	0.001 *
	12 months (T5)	11.94 ± 8.87	24.38 ± 3.94	−7.962	0.001 *
HAM-A:	initial (T1)	31.81 ± 5.71	27.41 ± 4.10	3.872	0.001 *
	1 month (T2)	26.45 ± 5.01	26.84 ± 4.68	−0.347	0.729
	3 months (T3)	21.16 ± 4.30	24.43 ± 4.19	−3.356	0.001 *
	6 months (T4)	16.00 ± 4.36	24.00 ± 3.19	−9.150	0.001 *
	12 months (T5)	11.35 ± 4.95	23.30 ± 4.01	−11.592	0.001 *
CGI–S:	baseline (T1)	5.72 ± 0.60	4.84 ± 0.74	5.649	0.001 *
CGI-I:	1 month (T2)	3.81 ± 0.46	3.79 ± 0.40	0.159	0.874
	3 months (T3)	3.00 ± 0.47	3.41 ± 0.49	−3.683	0.001 *
	6 months (T4)	2.37 ± 0.49	3.74 ± 2.39	−3.403	0.001 *
	12 months (T5)	2.02 ± 0.55	3.56 ± 0.71	−10.422	0.001 *

Legend: HAM-D = Hamilton Rating Scale for Depression; HAM-A = Hamilton Anxiety Rating Scale; CGI–S = Clinical Global Impressions—Severity Scale; CGI-I = Clinical Global Impressions—Improvement Scale; * = statistical significance, *p* < 0.05.

**Table 3:** Results of unifactorial ANOVA with repeated measurements based on RDD severity in both patient groups.

Disease Type	Patient Group	Sum of Squares	Df	Squared Mean Value	F	*p*	η^2^
RDD	Group A Genetically tested	13,098.184	4	3274.546	74.334	0.001	0.674
	Group B Not tested	730.082	4	182.521	21.218	0.001	0.358

Legend: RD = recurrent depression. Note: F = ANOVA; *p* < 0.05; η^2^ = effect size.

**Table 4:** Pairwise comparisons depending on the moment of assessment using HAM-D.

Disease Type	Patient Group	(A) HAM-D	(B) HAM-D	Mean of Differences (A–B)	Standard Error	*p*
RDD	Group A Genetically tested	Inclusion	1 month	4.97	2.23	0.321
			3 months	12.00 *	2.21	0.001
			6 months	17.27 *	2.21	0.001
			12 months	23.56 *	1.82	0.001
	Group B Not tested	Inclusion	1 month	1.10	0.50	0.366
			3 months	3.74 *	0.72	0.001
			6 months	4.30 *	0.64	0.001
			12 months	5.00 *	0.82	0.001

Legend: RD = recurrent depression; HAM-D = Hamilton Rating Scale for Depression. Note: * *p* < 0.01.

**Table 5:** Unifactorial ANOVA with repeated measurements for the analysis of anxiety.

Disease Type	Patient Group	Sum of Squares	Df	Squared Mean Value	F	*p*	η^2^
RDD	Group A Genetically tested	9774.13	4	2443.532	160.621	0.001	0.817
	Group B Not tested	514.79	4	128.697	18.875	0.001	0.332

Legend: RDD = recurrent depression. Note: Df = degrees of freedom, F = ANOVA, *p* < 0.01, η^2^ = effect size.

**Table 6:** Pairwise comparisons by time of HAM-A testing.

Disease Type	Patient Group	(A) HAM-A	(B) HAM-A	Mean of Differences (A–B)	Standard Error	*p*
RDD	Group A Genetically tested	Inclusion	1 month	5.35 *	0.71	0.001
			3 months	10.64 *	0.95	0.001
			6 months	15.81 *	1.05	0.001
			12 months	20.45 *	1.25	0.001
	Group B Not tested	Inclusion	1 month	0.56	0.43	1.000
			3 months	2.97 *	0.62	0.001
			6 months	3.41 *	0.50	0.001
			12 months	4.10 *	0.64	0.001

Legend: RD = recurrent depression; HAM-A = Hamilton Anxiety Rating Scale. Note: * *p* < 0.01.

## Data Availability

The original contributions presented in the study are included in the article, further inquiries can be directed to the corresponding author.
